# Community Group-Based Models of Medication Delivery: Applicability to Cardiovascular Diseases

**DOI:** 10.5334/gh.763

**Published:** 2021-05-05

**Authors:** Cordelia Kwon, Ruth Webster, Karla Santo, Emily Atkins

**Affiliations:** 1 College of Arts and Science, New York University, New York, US; 2 The George Institute for Global Health, UNSW, Sydney, AU; 3 Centre for Health Economics Research and Evaluation, UTS, Sydney, AU; 4 Academic Research Organization, Hospital Israelita Albert Einstein, São Paulo, BR; 5 Westmead Applied Research Centre, Westmead Clinical School, Faculty of Medicine and Health, The University of Sydney, Sydney, AU

**Keywords:** non-communicable diseases, adherence clubs, medication adherence, community models of care

## Abstract

The rising global burden of chronic non-communicable diseases (NCDs) has put a strain on healthcare systems globally, especially in low- and middle-income countries, which have seen disproportionate mortality rates due to non-communicable diseases. These deaths are in part due to challenges with medication adherence, which are compounded by lack of access to medication and weak community support systems. This paper aims to propose a potential solution using models of service delivery in HIV/AIDS, given the many similarities between NCD and HIV/AIDS. Models that have been particularly effective in HIV/AIDS are the community-based peer-support medication delivery groups: medication adherence clubs and community antiretroviral therapy (ART) groups. The positive outcomes from these models, including improved medication adherence and patient satisfaction, provide evidence for their potential success when applied to non-communicable diseases, particularly hypertension and cardiovascular disease.

## Introduction

Currently, healthcare systems are learning to adapt to a shifting disease landscape, in which the burden of communicable diseases is decreasing while, simultaneously, the global burden of non-communicable diseases (NCD) is rising [1]. In 2016, NCDs were responsible for 71% of the world’s 57 million deaths. Of those, 15 million deaths were premature, occurring in people aged between 30-70 years [1]. While this is a global problem, low- and middle-income countries (LMIC) are disproportionately affected, accounting for 78% of all NCD deaths [1]. The reasons for this high mortality rate include lack of access to medical care and lack of affordability of recommended medications, as well as medication non-adherence, a problem which is more prevalent in LMICs than high-income countries [2]. One demonstration of this problem in the management of atherosclerotic cardiovascular diseases (CVD), which is the leading cause of death among NCDs, was detailed in the PURE study. The PURE study showed that the use of cardiovascular preventive medications (statins, antiplatelets and blood pressure lowering drugs) tends to decrease in line with a decrease in the country’s income level [3]. In low-income countries, less than 10% of participants, all of whom had a history of heart disease or stroke, were taking the four major cardiovascular secondary prevention drugs [3]. There was also high variation between urban and rural areas for LMICs, with those in urban areas having higher treatment coverage rates, showing that access is a significant part of the problem [3]. Whether it is due to lack of access, lack of availability, or human error (such as forgetfulness), when patients do not adhere to their preventive medications, there is an associated two-fold increase in the rate of CVD events compared to those who are adherent [4]. Therefore, cost-effective, scalable solutions are needed to improve access and adherence to recommended CVD preventive medications in LMIC in order to reduce complications related to CVD, and thus the burden on the healthcare systems.

Many of the same healthcare systems that are now dealing with this rising prevalence of CVD, including risk factors such as diabetes and hypertension, and the need to improve medication adherence to preventive medications, have already put in healthcare structures to deal with the parallel crisis of HIV/AIDS, leading to significant interest in leveraging this infrastructure to help manage the rising burden of CVD as well [5]. This is true for many countries in sub-Saharan Africa, which have the highest age-standardized mortality rates for both HIV/AIDS and NCDs [5]. In this geographical area, HIV/AIDS management has improved in the last two decades and now the percentage of people accessing anti-retroviral therapy (ART) is over 50% [6]. On the other hand, only 10% of patients with established CVD are taking medication in low-income countries [3] with similarly low rates for CVD risk factors, such as hypertension [7]. These data show that the success in improving access to HIV/AIDS medications has not yet been transferred to NCD. Besides the double burden of HIV/AIDS and NCD in sub-Saharan Africa, there are many shared barriers to care in these two diseases contexts. Both HIV/AIDS and CVD requires consistent medication regimes; ongoing, lifelong access to primary health care; healthy living and self-management, including high adherence to continued self-monitoring and to medications. In addition to patient-level barriers, the continued management of HIV/AIDS as well as CVD patients places a large economic burden on the clinics that must support them [5]. To address these latter concerns, there is evidence that decentralizing clinical service and moving care from facilities into the community would decrease the clinics’ costs [6]. Effective community-based interventions have already been developed and evaluated for HIV/AIDS. One such effective intervention is the community-based medication delivery strategy that relies on a club or group setting to deliver the medications to the patients and promote adherence. These community-based medication delivery strategies have been shown to have statistically significant increases in retention in care, lower costs, and shorter waiting times in clinics involved in the management of HIV/AIDS patients [8].

The aim of this paper is to present a hypothesis generating narrative review of the literature to describe the factors that led to the success of these community-based medication delivery strategies at national healthcare system, clinic, and patient levels in the HIV/AIDS setting, and to discuss whether such interventions may be transferable to the CVD setting in LMIC, specifically in patients with conditions that are amenable to decentralized management in the community including stable patients with coronary heart disease or stroke, or risk factors such as hypertension and diabetes.

## Methods

We used a hierarchical search strategy to identify relevant articles related specifically to medication delivery/adherence clubs involving club or group settings, including:

1. Pubmed search including the following search terms: ‘medication adherence clubs,’ ‘community ART groups AND HIV/AIDS,’ and ‘community health service delivery AND (non-communicable OR chronic) diseases.’
2. Searching the reference lists of identified articles from the pubmed search.
3. Discussion with experts currently working in this area to identify any articles that may have been missed.

Identified articles were collated and data was extracted on program elements, efficacy data, and any description around factors that facilitated effectiveness and sustainability of these programs. Results were collated and summarized for key themes and messages and evaluated for applicability to the management of stable cardiovascular disease and its risk factors.

## Results

Eighteen articles of relevance were identified, covering six countries. Many studies were closely linked to each other in evaluating the same program, or in evaluating programs that were established as a result of the successful implementation of a similar program elsewhere. A summary of the studies and their connections to each other is provided below.

### South Africa

- Retrospective cohort study [8] with associated health economic analysis [9] of a pilot program in Khayelitsha, South Africa, evaluating effectiveness and cost-effectiveness of the pilot program.
- Retrospective cohort study [10] in the Western Cape, South Africa (scaleup of the pilot study in Khayelitsha), evaluating effectiveness of the scaleup program.
- Analytical cross-sectional study [11] in Eden, South Africa (part of Western Cape scaleup), evaluating patients’ satisfaction with adherence clubs and relationship to treatment adehrence.
- Qualitative study [12] in Khayelitsha and Gugulethu, South Africa (part of the Western Cape scaleup), exploring patients’ experiences with adherence clubs.
- Five papers outlining a broad program of realist theory testing case studies [13,14,15,16,17] in Western Cape, South Africa, evaluating how and why adherence clubs work.
- A scoping review [18] of sustainability factors of the scaleup of medication clubs in the Western Cape.

### Mozambique

- A cohort study in Tete, Mozambique [19], with an associated qualitative study [20].

### Malawi

- A qualitative study in Thyolo, Malawi [21], evaluating benefits and limitations of comunity ART groups (this program grew out of the successful program implemented in Tete, Mozambique).

### Zimbabwe

- A qualitative study in Zimbabwe [22], evaluating the perceived effects of community ART refill groups (this program grew out of the successful program implemented in Tete, Mozambique).

### Haiti

- Descriptive case study [23] of an adapted community ART group model in Haiti (this program was based on the successful program implemented in Tete, Mozambique).

### Kenya

- Retrospective descriptive study [24] in Kibera, Kenya (this program trialled expansion of the club model from South Africa to include a mixed chronic disease population).
- Qualitative study [25] evaluating the acceptability of integrated medication adherence clubs in Kibera, Kenya.

### Sub-saharan Africa

- A review article of community-based models for HIV healthcare delivery in South Africa, Democratic Republic of Congo, Malawi, and Mozambique (this paper summarised results from several other papers)

## Community-Based Medication Delivery Strategies in HIV/AIDS

Broader community-based medication delivery strategies in HIV/AIDS may include a variety of initiatives, including medical appointment spacing and fast-track drug refilling, community antiretroviral therapy (ART) distribution points, medication adherence clubs (MACs), and community ART groups (CAGs) [26], with the last two approaches being of key interest to this paper. All these strategies target stable patients who have a degree of immune recovery or are virologically suppressed, aiming to decentralize care and separate the dispensing of ART refills from clinical consultation. This decentralization allows patients to access their medications without having to make frequent long trips to the clinic and wait long hours for a clinical evaluation and to collect the medication. In addition to easier access to the medication, community ART groups (CAGs), and medication adherence clubs (MACs) are unique for having an element of adherence support from peers. There are some key differences between these two approaches. CAGs are patient-led, and group members take turns picking up and distributing the medication, and the group provides adherence support and monitors treatment outcomes. Clinical consultation for members occurs either as a group in-clinic or when members need refills. MACs are led by a lay health worker or peer counselor, who distributes medications and monitors symptoms, as well as providing peer support when distributing medication [26]. In terms of implementation, it is important to consider the community’s human resources before deciding between these two approaches. Beyond that, these two approaches function similarly and have similar positive outcomes, with the shared goal of these groups being to improve adherence to medications by reducing burdens for patients and the healthcare system, increase retention in care, and lower service-provider costs.

## Effectiveness of Community-Based Medication Delivery Clubs/Groups

The two key programs were those from South Africa and Mozambique, with other projects utilizing the successes of these programs to establish their own programs. Two papers reported lower mortality rates with both MACs and CAGs in both South Africa and Mozambique, respectively [8,19], as well as greater retention in care, lower costs to the clinic [9,19], and higher patient satisfaction (see Table 1 for summarized data from these programs) [11]. The success of these initial programs has led to national scale-up in both South Africa and Mozambique, with 18,000 patients in South Africa and 17,000 in Mozambique being treated with these approaches in 2014 [27]. In qualitative studies, patients were satisfied with the club model, especially in regard to their peer support and time-saving qualities [12,20,21].

**Table 1:** Summarized Outcomes of Medication Adherence Clubs and Community ART Groups.

	Medication Adherence Clubs	Community ART Groups

*Country*	South Africa	Mozambique
*Study Design*	Cohort analysis and comparison club care vs. usual care	Retrospective cohort study (survival analysis)
*Duration of follow-up (median per person)*	39 months [38–39] for those study participants (n = 502)	19 months [10–29] (n = 5729)
*Mortality*	<1% at 40 months	2.1 per 100 person-years
*Loss to follow-up*	2.8% at 40 months	0.1 per 100 person-years
Retention in care in program*	97% at 40 months in club (n = 502) vs. 85% in usual care (n = 2327)	97.7% at 12 months, 96.0% at 24 months, 93.4% at 36 months, 91.8% at 48 months
*Cost per patient*	$58 p/patient year in club vs. $109 in usual care	49.6% reduction of clinic visits with reduction of 62% in ART refill visits
*Patients currently enrolled (% active ART cohort)*	5909 (23%) Roll-out in Cape Town (Western Cape): 18,719—19% of overall ART cohort (98,233)	8181 (50%) 17,272 patients in CAGs country wide (October 2013)

* Retention in care: total number of patients on ART care followed in the program (excluding those who are transferred out). Table adapted from Bemelmans et al. (2014).

The Mozambique program in particular inspired two similar programs to be implemented in Southern Haiti and Zimbabwe [22,23]. Both of these programs have reported high rates of satisfaction from both patients and care providers, and have empowered patients in their own HIV care [22,23].

## Sustainability Factors

**Figure 1:**
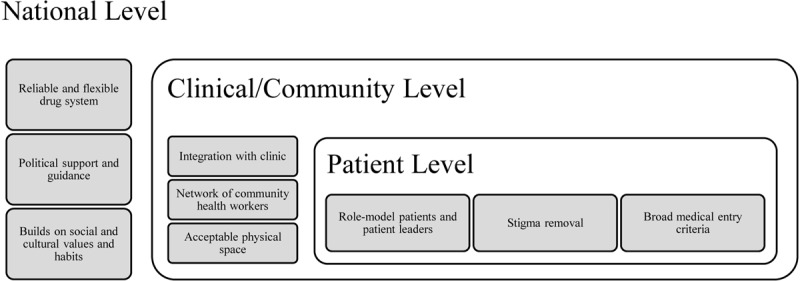
Groups of Sustainability Factors.

The success of these clubs shows the strength of community-based care. However, in addition to the effective structure, there are external factors that influence the success of the program (Figure 1). These factors exist at macro, meso, and micro (i.e., national, community, and patient) levels, but each level affects the others in multi-faceted ways. One essential aspect when considering implementation of these clubs is a national drug system that can consistently supply medicines to patients [28]. Of note, medication for HIV/AIDS is often provided for free in donor-led programs, which may not be the case for other diseases. Therefore, generalizability of the success of these clubs—when patients need to pay for medications—is unclear. Also, there must be a national buy-in from policy makers, even before scale-up, to aid implementation and help support the drug system. For this national buy-in to be in place, it is essential to ensure that these clubs are socially acceptable and built on cultural values and structures. On a community level, an active conversation between the clinic and the club is essential in ensuring that there are bilateral referrals. Patients must be able to fluidly move between clinic and club as their conditions worsen or improve [14,18]. There also must be a network of community health workers in place. For MACs, they are needed to run the clubs and ensure proper education. The burden for CAGs is less, as these are patient-led clubs; however, community health workers are often still necessary to train the participants in proper adherence and check-up behavior [12]. Finally, the physical space of the club, whether it be in the community or in a clinic space, should be comfortable and free of stigma for the patients. The club space directly impacts on the patient-level factor, as patients must feel comfortable in the club by being surrounded by peers with similar disease contexts, and peer leaders who are empathic to their needs [14,18]. In addition, it is also important to ensure that the medical criteria for patients under consideration for entry into the club is not so strict that it ultimately excludes most community members [12].

## Applicability to Non-Communicable Diseases

These sustainability factors provide a framework when considering how the HIV/AIDS club model can be applied to NCD, especially CVD, given the high degree of similarity between these two epidemics. Many of the barriers to ART adherence also apply to CVD medication adherence, particularly those barriers that MACs and CAGs address. These include drug supply/procurement, referrals and linkages, community involvement, and importantly, lack of adherence support. Because of all these shared similarities in barriers to care, the medication delivery club model in place for HIV/AIDS could help address many of those barriers in CVD [5]. Not only are the barriers and challenges similar, but in the places where HIV/AIDS clubs are already in place, the resources and structures are already functional and effective [29]. For example, a wide network of community health workers already trained in HIV/AIDS club management could readily be trained in CVD club management, and thus a potential sustainability factor is already more accessible. Therefore, the external factors that contributed to the success of HIV/AIDS medication delivery clubs would also aid the success of potential NCD-focused clubs, and thus should be considered when interventions are being developed.

### Feasibility challenges

However, there are also several factors that differ between the context of HIV/AIDS and management of atherosclerotic CVD and its risk factors, which may impact the likely transferability of this effectiveness. Firstly, there is significant global investment from donor agencies in the management of HIV/AIDS, which is largely responsible for the successful coverage of ART via provision of free drugs. Despite growing acknowledgement of the epidemic of CVD (amongst other NCDs) similar interest and investment has not yet occurred, meaning that the majority of patients in LMIC must pay for CVD medications out of pocket. Additionally, untreated HIV rapidly becomes symptomatic, which is likely to positively impact patients’ likelihood of taking medication, whereas hypertension or uncontrolled diabetes may well be asymptomatic. Even if a patient has already had a heart attack or stroke, they may think they are now ‘cured’ because they are out of the hospital, and therefore not prioritize all the medications they are prescribed. Additionally, due to extensive education programs over the last few decades, awareness of HIV/AIDS is high amongst highly prevalent countries, with commensurate awareness still lacking around NCDs. However, this last point may actually argue in favor of peer support clubs to improve support and education for such patients.

### Clinic-based integrated care delivery

There is some evidence that clinic-based approaches that adapt or integrate the care of patients with NCD into already existing structures for patients with HIV/AIDS are effective. In Ethiopia, an HIV/AIDS clinic adapted registers, charts, flow sheets, and training of community health workers and peer educators for diabetes, leading to an increase in quality of care after six months for patients with diabetes [5]. In Cambodia, MSF and the government piloted two ‘chronic disease clinics’ (as opposed to disease specific clinics) for integrated care for HIV, diabetes, and hypertension, which led to high rates of retention for all the diseases—between 70%–90% [5]. Even though these are clinic-based interventions, rather than community-based, it demonstrates feasibility of the concept.

### Community-based integrated medication adherence clubs (HIV/AIDS+)

To date, there is one relevant case study, conducted in Kibera, Kenya, where integrated care was applied to medication adherence clubs in a LMIC. This adherence club integrated hypertension, diabetes mellitus, and/or HIV/AIDS patients [24]. A total of 2,208 consultations originally done in the clinic were done in the community, saving the clinic the burden of routine check-ups and refills in terms of both time and money [24]. Only 2% of the patients required referral back to the clinic due to worsening of the health condition, and only 3.5% were lost to follow-up [24], showing the success of these clubs when simple medication processes were shifted to the community. A qualitative study of this same intervention found that the overall acceptability of the club was high and that many patients perceived clear benefits from it, including time-saving qualities, convenience, and peer support [25].

In the Kenya study, stigmatization was not found as a barrier to adherence for club members. However, stigma towards diseases can vary widely, with diseases such as HIV/AIDS and diabetes facing higher amounts of discrimination than CVD [5]. Furthermore, there is some evidence that integrated care may lead to discomfort of patients, if they are treated with patients whose diseases differ from their own [13]. In general, these clubs fulfill their function best if the patients feel as though they can relate to each other’s experiences and lifestyles. This feeling of comfort could potentially be hindered by patients with varying diseases being integrated into one club.

### Community-based clubs for cardiovascular disease (or risk factors)

A recent paper reported the implementation of a community peer-support group that is solely focused on adherence to antihypertensive medication, and is looking to show the feasibility of adapting either MAC or CAG approaches, depending on the context, to the NCD setting. However, results as to its success or otherwise are not yet available [30].

Overall, although there are promising reports, there is a lack of sufficient evidence as yet that MACs and CAGs are readily transferable to the CVD (or any other NCD) setting, and like all interventions, context is key; such clubs may work in some areas and not others.

## Recommendations

Given the many similarities between NCD and HIV/AIDS treatment and barriers to adherence, there is evidence that effective community-based care interventions in HIV/AIDS may be feasible to be delivered to patients with NCD. A particularly successful model in HIV/AIDS has been community-based medication delivery clubs/groups, which not only provide medication and routine check-ups at more convenient times and places to the patients, but also provide additional peer support. These community-based medication delivery clubs/groups have been shown to increase retention, lower loss to follow up, decrease mortality, and cost significantly less than traditional services delivered within the clinic setting. Promising results have also been reported with integrated care, where patients with diabetes and hypertension and those with HIV/AIDS are managed in the same settings, be it a clinic or community setting. However, there is also evidence that single-disease clubs are more ideal, due to patients being more comfortable within a group of people with similar lived experiences.

Based on the findings discussed in this paper, there is potential for community-based medication delivery clubs/groups—which are ideally solely focused on supporting people with NCD—to improve outcomes, similar to what has happened in the HIV/AIDS context. Implementing more community-based healthcare models, such as the community-based medication delivery clubs/groups, for different disease areas may, therefore, help to decrease the strain on healthcare systems in LMIC, which will be particularly impactful given the high prevalence of NCD in these countries. Additional research is needed to demonstrate this predicted benefit and any additional sustainability factors needed in the NCD context.

## Abbreviations

NCD: non-communicable diseases; LMIC: low- and middle-income country; ART: antiretroviral therapy; MAC: medication adherence club; CAG: community ART group; CVD: cardiovascular disease; HIV/AIDS: human immune-deficiency virus/acquired immunodeficiency syndrome.
